# The Science and Art of Midwifery

**Published:** 1886-01

**Authors:** 


**The Science and Art of Midwifery.** By WM. THOS. LUSK, A. M., M. D., Professor of Obstetrics and the Diseases of Women and Children in the Bellevue Hospital Medical College; Consulting Physician to the Maternity Hospital, Gynæcologist to the Bellevue Hospital, etc., etc. New edition, revised and enlarged, with numerous illustrations. New York: D. Appleton & Co. 1885.

It is only about three years ago that the first edition of this work appeared. That so soon another edition is called for, is an indication of the favor with which this great work has been received. Indeed, the appreciation of it has not been confined to the English-speaking countries, as it has been already translated into Spanish, French and Italian. The author has availed himself of the opportunity for revision, and has made some changes in the text. It now presents all that modern science has contributed to the art of midwifery. He dwells upon the triumphs of the modern doctrine concerning the nature of puerperal fever. The illustrations in the book are all excellent. Taken as a whole, the book is one of the most complete and reliable ones extant, and we cannot too heartily commend it.

